# Taurine does not affect the composition, diversity, or metabolism of human colonic microbiota simulated in a single-batch fermentation system

**DOI:** 10.1371/journal.pone.0180991

**Published:** 2017-07-10

**Authors:** Kengo Sasaki, Daisuke Sasaki, Naoko Okai, Kosei Tanaka, Ryohei Nomoto, Itsuko Fukuda, Ken-ichi Yoshida, Akihiko Kondo, Ro Osawa

**Affiliations:** 1 Graduate School of Science, Technology and Innovation, Kobe University, 1–1 Rokkodai-cho, Nada-ku, Kobe, Hyogo, Japan; 2 Organization of Advanced Science and Technology, Kobe University, 1–1 Rokkodai-cho, Nada-ku, Kobe, Hyogo, Japan; 3 Department of Bioresource Science, Graduate School of Agricultural Science, Kobe University, 1–1 Rokkodai-cho, Nada-ku, Kobe, Hyogo, Japan; 4 Research Center for Food Safety and Security, Graduate School of Agricultural Science, Kobe University, 1–1 Rokkodai-cho, Nada-ku, Kobe, Hyogo, Japan; 5 RIKEN Center for Sustainable Resource Science, 1-7-22 Suehiro-cho, Tsurumi-ku, Yokohama, Kanagawa, Japan; "INSERM", FRANCE

## Abstract

Accumulating evidence suggests that dietary taurine (2-aminoethanesulfonic acid) exerts beneficial anti-inflammatory effects in the large intestine. In this study, we investigated the possible impact of taurine on human colonic microbiota using our single-batch fermentation system (Kobe University Human Intestinal Microbiota Model; KUHIMM). Fecal samples from eight humans were individually cultivated with and without taurine in the KUHIMM. The results showed that taurine remained largely undegraded after 30 h of culturing in the absence of oxygen, although some 83% of the taurine was degraded after 30 h of culturing under aerobic conditions. Diversity in bacterial species in the cultures was analyzed by 16S rRNA gene sequencing, revealing that taurine caused no significant change in the diversity of the microbiota; both operational taxonomic unit and Shannon-Wiener index of the cultures were comparable to those of the respective source fecal samples. In addition, principal coordinate analysis indicated that taurine did not alter the composition of bacterial species, since the 16S rRNA gene profile of bacterial species in the original fecal sample was maintained in each of the cultures with and without taurine. Furthermore, metabolomic analysis revealed that taurine did not affect the composition of short-chain fatty acids produced in the cultures. These results, under these controlled but artificial conditions, suggested that the beneficial anti-inflammatory effects of dietary taurine in the large intestine are independent of the intestinal microbiota. We infer that dietary taurine may act directly in the large intestine to exert anti-inflammatory effects.

## Introduction

Taurine (2-aminoethanesulfonic acid) is a semi-essential amino acid and one of the most abundant free amino acids in mammalian tissues such as heart and brain [1]. Taurine is known to play a wide range of critical roles in both human and animal physiology, including functions in antioxidation, osmoregulation, bile acid conjugation, regulation of blood pressure, maintenance of retinal and cardiac function, regulation of neuroendocrine activity, and prevention and treatment of fatty liver disease [2–8]. In addition, taurine may have anti-inflammatory effects, as demonstrated by the ability of dietary taurine to attenuate artificially induced colitis in mice [9]. Consistent with this role, human intestinal epithelial Caco-2 cells grown in the presence of taurine exhibit enhanced expression of genes encoding proteins that protect against inflammation [10].

Recently, Yu et al. [11] reported a beneficial effect of dietary taurine in conjunction with gut microbiota; specifically, dietary taurine inhibited the growth of harmful bacteria, increasing the production of short-chain fatty acids (SCFA) in mouse feces. In contrast, Ridlon et al. [12] suggested that certain intestinal bacteria convert taurine-conjugated bile acid to hydrogen sulfide, a genotoxic compound. The above claims were, however, based exclusively on experiments performed in animal models, the gut microbiota of which is markedly different from that of humans [13,14]. Moreover, the correlation between gut microbiota and taurine itself remains unclear; although the interactions between gut microbiota and taurocholic acid/bile acid have been investigated [12,15,16]. Given both ethical and funding considerations [17,18], few studies on the role of dietary taurine have employed human intervention trials. In order to circumvent this limitation, we have developed a single-batch anaerobic culturing system that simulates the human colonic microbiota both metagenomically and metabolically. This system (hereafter referred to as the Kobe University Human Intestinal Microbiota Model, or KUHIMM) was designed to monitor transitional changes in the composition of bacterial groups and their metabolites by analyses combining quantitative PCR, next-generation sequencing (NGS), and high-performance liquid chromatography (HPLC). The KUHIMM has been successfully used to reproduce the prebiotic effects of various oligosaccharides observed in human intervention trials [19]. We therefore used the KUHIMM to investigate the effects of taurine on the human colonic microbiota.

## Materials and methods

### Fecal samples

We obtained fresh fecal samples from 8 healthy human volunteers who had provided written informed consents prior to specimen collection. Volunteers included M39 (male, age 39, Japanese), M38 (male, age 38, Japanese), F40 (female, age 40, Japanese), M43 (male, age 43, Japanese), M60 (male, age 60, Japanese), F62 (female, age 62, Japanese), M25 (male, age 25, Japanese), and M34 (male, age 34, Japanese). None of these individuals had a history of antibiotic treatment in the 6 months prior to the study. Immediately following collection, each fecal sample was stored in an anaerobic culture swab (212550 BD BBL Culture Swab; Becton, Dickinson and Company, New Jersey, USA) and used within 24 h. All experimental protocols were approved by the institutional ethics review board at Kobe University, and followed the guidelines approved by the Medical Ethics Committee at Kobe University.

### Initiation of the KUHIMM

We used a small-scale multi-channel fermentor (Bio Jr.8; ABLE, Tokyo, Japan) composed of up to eight parallel and independent anaerobic culturing vessels, as described by Takagi et al. [19]. Thus, many independent experiments can be run by using each vessel. Each vessel contained 100 mL of autoclaved (115°C for 15 min) Gifu anaerobic medium (GAM [Code 05422]; Nissui Pharmaceutical Co, Tokyo, Japan), with the initial pH adjusted to 6.5. The medium was held at 37°C with constant stirring at 300 rpm under anaerobic conditions; anaerobiosis was maintained by continuous in-flow (15 mL/min) of a N_2_:CO_2_ (80:20) gas blend that was filter-sterilized through a 0.2-μm PTFE membrane (Pall Corporation, IL, USA) starting before the fermentation initiation and continuing during fermentation. Prior to use in inoculation, each fecal sample was suspended in 2 mL of 0.1 M phosphate buffer (pH 6.5, consisting of a 2:1 mixture of NaH_2_PO_4_ and 0.1M Na_2_HPO_4_) supplemented with 1% L-ascorbic acid (Wako Pure Chemical Industries, Osaka, Japan). Each of a set of 2 vessels was inoculated with 100 μL per vessel of the above fecal suspension to initiate the KUHIMM.

### Addition of taurine to the KUHIMM and culture sampling

Immediately after fecal inoculation, one of each set of vessels was supplemented with taurine (generously provided by Mitsui Chemical Inc.) to a final concentration of 10 mM; the remaining 1 of each set of 2 vessels received no supplementation. The initial concentration of taurine was set to 10 mM based on the assumption that an adult human would ingest 3 g of taurine, the upper limit for daily supplemental intake [20], as part of a daily diet of 3 L of food and water per day (3 g per 3 L corresponds to a concentration of ca. 10 mM taurine). Following initiation of fermentation, samples of approximately 1 mL/time point were collected from each fermentation culture at 0, 6, 9, 12, 24, and 30 h for SCFA analyses and at 30 h only for real-time PCR, taurine measurements, and sequencing. The pH of the cultures was checked at each time point, and adjusted back to pH 6.5 only when the pH of the medium exceeded 6.6. The collected culture samples were stored at –20°C until use.

### Microbial DNA extraction

Extraction of microbial DNA from suspended fecal samples and cultured microbiota was performed as described previously [19]. In short, a 200-μL aliquot of each culture or suspended fecal sample was transferred to a sterile bead-beating tube containing 300 mg of 0.1-mm-diameter glass beads. This mixture was combined with 500 μL of TE (10 mM TrisHCl, 1 mM EDTA, pH8.0) -saturated phenol, 250 μL of lysis buffer, and 50 μL of 10% (wt/vol) sodium dodecyl sulfate. The resulting mixture was then shaken vigorously for 30 s at 5.0 m/s in a FastPrep-24 instrument (MP Biomedicals SARL, Illkirch, France). After centrifugation at 20,000 g for 5 min, the upper layer was transferred to a new tube. The nucleic acid-containing aqueous phase then was extracted using 400 μL of phenol-chloroform-isoamyl alcohol (25:24:1) and centrifuged at 20,000 g for 5 min. The upper layer was transferred to a new tube and then DNA was precipitated by adding 275 μL of isopropyl alcohol and a 1/10 volume of 3 M sodium acetate, mixing, and placing the tube at –20°C for 10–15 min. The pellet was washed with 70% ethanol by centrifugation at 20,000 g for 5 min, and then dried under vacuum. DNA was subsequently dissolved in TE buffer.

### Illumina library generation

We amplified the V3-V4 region of the bacterial 16S rRNA genes using S-D-Bact-0341-b-S-17 (5’-CCTACGGGNGGCWGCAG-3’) and S-D-Bact-0785-a-A-21 (5’-GACTACHVGGGTATCTAATCC-3’) [21], where the N, W, H, and V symbols correspond to degenerate nucleotides A/C/G/T, A/T, A/C/T, and A/C/G, respectively. Illumina adapter overhang nucleotide sequences were added to the gene-specific sequences. The PCR reaction and preparation of the amplicon pool was performed according to the manufacturer’s instructions. Each PCR reaction used 12.5 ng of template DNA, along with 200 nM of each primer and 12.5 μL of KAPA HiFi HotStar ReadyMix (KAPA Biosystems, Massachusetts, USA). PCR reaction conditions were as follows: initial denaturation at 95°C for 3 min; 25 cycles at 95°C for 30 s, 55°C for 30 s, and 72°C for 30 s; and a final extension at 72°C for 5 min. Amplicons were first purified using the Agencourt AMPure XP beads (Beckman Coulter, Inc., California, USA). To normalize the sample amplicons, DNA concentrations in the PCR products were measured using the Qubit ds DNA HS Assay Kit (Thermo Fisher Scientific, MA, USA). The pooled 16S rRNA gene products (5 nM) (along with an internal control (PhiX control V3; Illumina, Tokyo, Japan)) was subjected to paired-end sequencing using a MiSeq sequencer (Illumina) with a 600-cycle MiSeq reagent kit (Illumina). The PhiX sequences were removed and paired-end reads with Q scores of ≥20 were joined using the software package QIIME version 1.9.1 [22]. The UCLUST algorithm [23] was used to cluster filtered sequences into Operational Taxonomic Units (OTUs) based on a 97% similarity threshold. Chimeric sequences were detected and excluded from the library using USEARCH [23]. Representative sequences from each OTU were taxonomically classified via the GreenGenes taxonomic database using the Ribosomal Database Project (RDP) Classifier [24]. OTUs were used for alpha-diversity estimation of Shannon-Wiener diversity. Principal coordinate analysis (PCoA) was conducted using OTU information from each sample and calculated based on unweighted UniFrac distances using QIIME. All the raw sequence data generated in this study have been deposited in MG-RAST as “Single Batch Fermentation System Simulating Human Colonic Microbiota_Taurine” under the accession numbers 4721803.3–4721826.3.

### Real-time PCR

We performed real-time PCR to quantify total bacterial growth during culturing using a TP700 Thermal Cycler Dice Real Time System Lite (Takara Bio, Ohtsu, Japan) with a primer set targeting all eubacteria. Each PCR reaction was performed in duplicate in a volume of 20 μL containing 2 μL template DNA solution, 10 μL THUNDERBIRD^TM^ SYBR^®^ qPCR Mix (Toyobo, Osaka, Japan), 7.2 μL distilled water, and 200 nM each primer. Amplification was performed with the following temperature profiles: one cycle at 95°C for 3 min, followed by 38 cycles at 95°C for 30 s and 55°C for 30 s. Melt curve analyses were generated by heating the PCR mixtures from 60 to 95°C (with increase of 1°C per cycle of 20 s) with simultaneous measurements of the SYBR Green I signal intensities. Quantification was performed by using standard curves generated from known concentrations of PCR fragments of the 16S rRNA genes.

### Chemical analyses

Concentrations of taurine were measured with the Prominence Amino Acid Analysis System (Shimadzu) according to the manufacturer’s instructions [25]. In short, each sample (10 μL) was mixed with 990 μL of 0.2 M sodium citrate buffer (pH 2.2) and the concentration of taurine in the aliquot was determined based on standard curves generated using taurine. In addition, levels of SCFAs such as acetate, propionate, butyrate, lactate, and succinate were measured using a high-performance liquid chromatograph (HPLC) (Shimadzu, Kyoto, Japan) equipped with an Aminex HPX-87H column (Bio-Rad Laboratories, Hercules, CA, USA) and RID-10A refractive index detector (Shimadzu); the HPLC was operated at 65°C using 5 mM H_2_SO_4_ as the mobile phase, with a flow rate of 0.6 mL/min. In addition, ammonium concentrations were measured with the Ammonium Reagent Set (HACH1295, Hach, Loveland, CO, USA).

### Aerobic cultivation

As will be shown below, taurine added to the KUHIMM was intact even after a 30-h-long anaerobic incubation. In contrast, others [26] have reported that taurine can be utilized by *Escherichia coli* growing under aerobic condition. To address this distinction, we conducted an additional experiment as follows. An aliquot (100 μL-) of each human fecal suspension prepared from 4 of the above human subjects was propagated aerobically for 30 h at 37°C and 150 rpm in 100 mL GAM medium containing 10 mM taurine. Aliquots of approximately 2 mL of each culture were sampled at 0 and 30 h after the initiation of cultivation and then analyzed for taurine by HPLC, as described above.

### Statistics and data analysis

Microbial diversity was assessed using the Shannon-Wiener diversity index (H’ = ‒∑[(*p*i)ln(*p*i)], where *p*i is the proportion of total species represented by species i [27]. The Shannon-Wiener diversity index was calculated using the QIIME software package. Data for the OTUs (roughly species-level classification) and Shannon-Wiener index were compared between fecal samples and cultures or between cultures with taurine containing taurine and those lacking taurine, by using the Kruskal-Wallis test [28] of the JMP software package (version 12; Institute, Tokyo, Japan). *P* values of less than 0.05 were considered significant.

## Results

### No utilization of taurine under anaerobic condition

**Fig 1:**
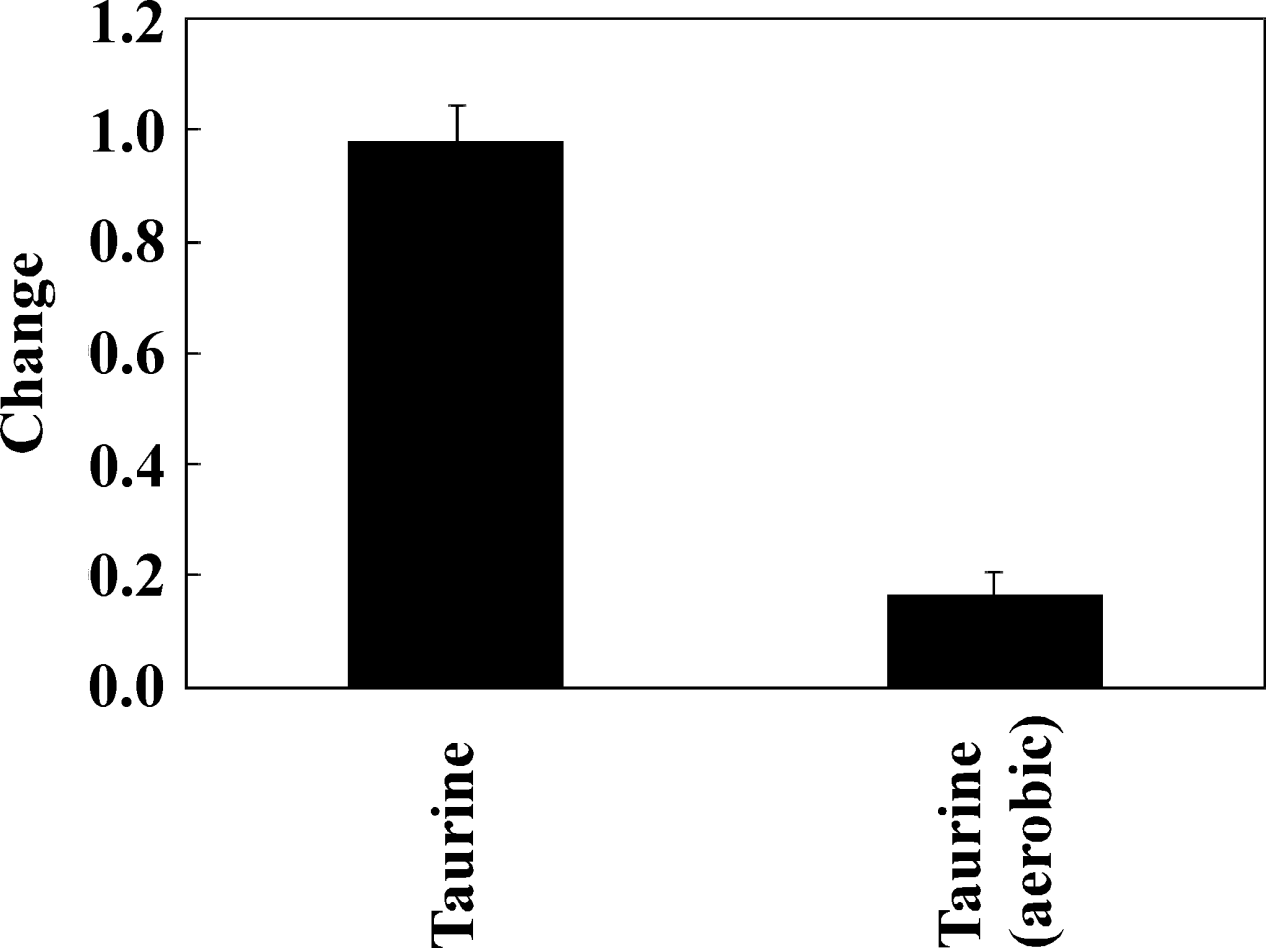
Changes in concentrations of taurine in the Kobe University Human Intestinal Microbiota Model (KUHIMM) cultures. “Change” indicates the ratio of the concentration at 30 h after the initiation of fermentation to the starting concentration (about 10 mM). Fermentation was initiated by inoculating separate cultures with one of each of the human fecal samples (M39, M38, F40, M43, M60, F62, M25, and M34). In addition, changes in taurine concentration were investigated under aerobic conditions in the KUHIMM following inoculation of 4 separate cultures with one of each of 4 of the human fecal samples (M39, M38, F40, and M34).

First, we checked whether the added taurine was consumed by the microbiota in the human large intestine as modeled using the KUHIMM. Taurine was added at 10 mM to each of the KUHIMM cultures that had been inoculated using one of the 8 human fecal samples (M39, M38, F40, M43, M60, F62, M25, or M34) and grown under anaerobic conditions. Greater than 95% of the added taurine remained at 30 h after the initiation of fermentation (Fig 1), indicating that most of the taurine was not consumed in the absence of oxygen.

### Effect of taurine on human colonic microbiota

**Fig 2:**
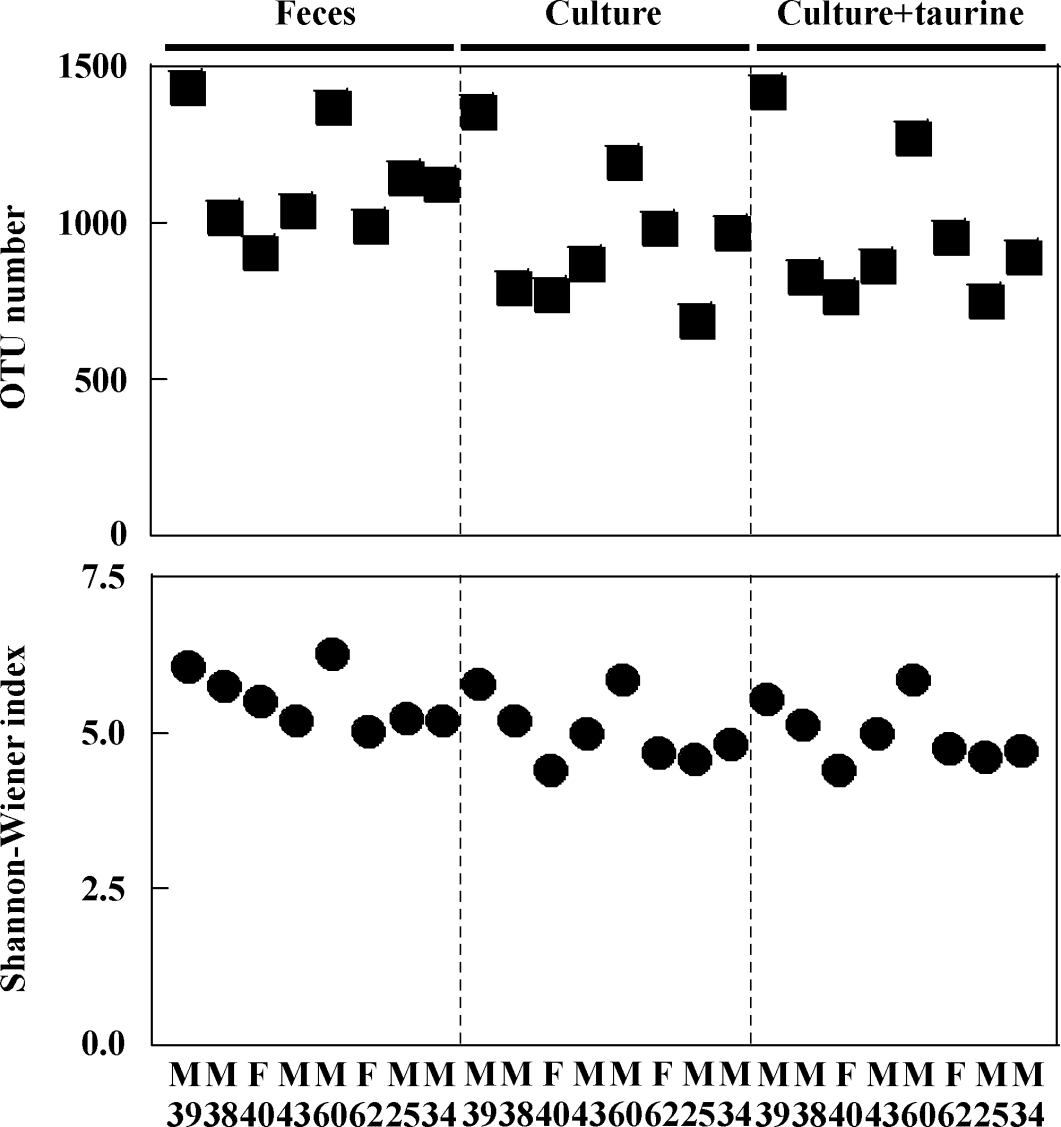
Comparison of operational taxonomic unit (OTU) number and bacterial diversity (Shannon-Wiener index) among original fecal samples and respective KUHIMM cultures grown in the absence or presence of taurine following inoculation with one of each of the fecal samples from 8 human subjects (M39, M38, F40, M43, M60, F62, M25, and M34).

**Fig 3:**
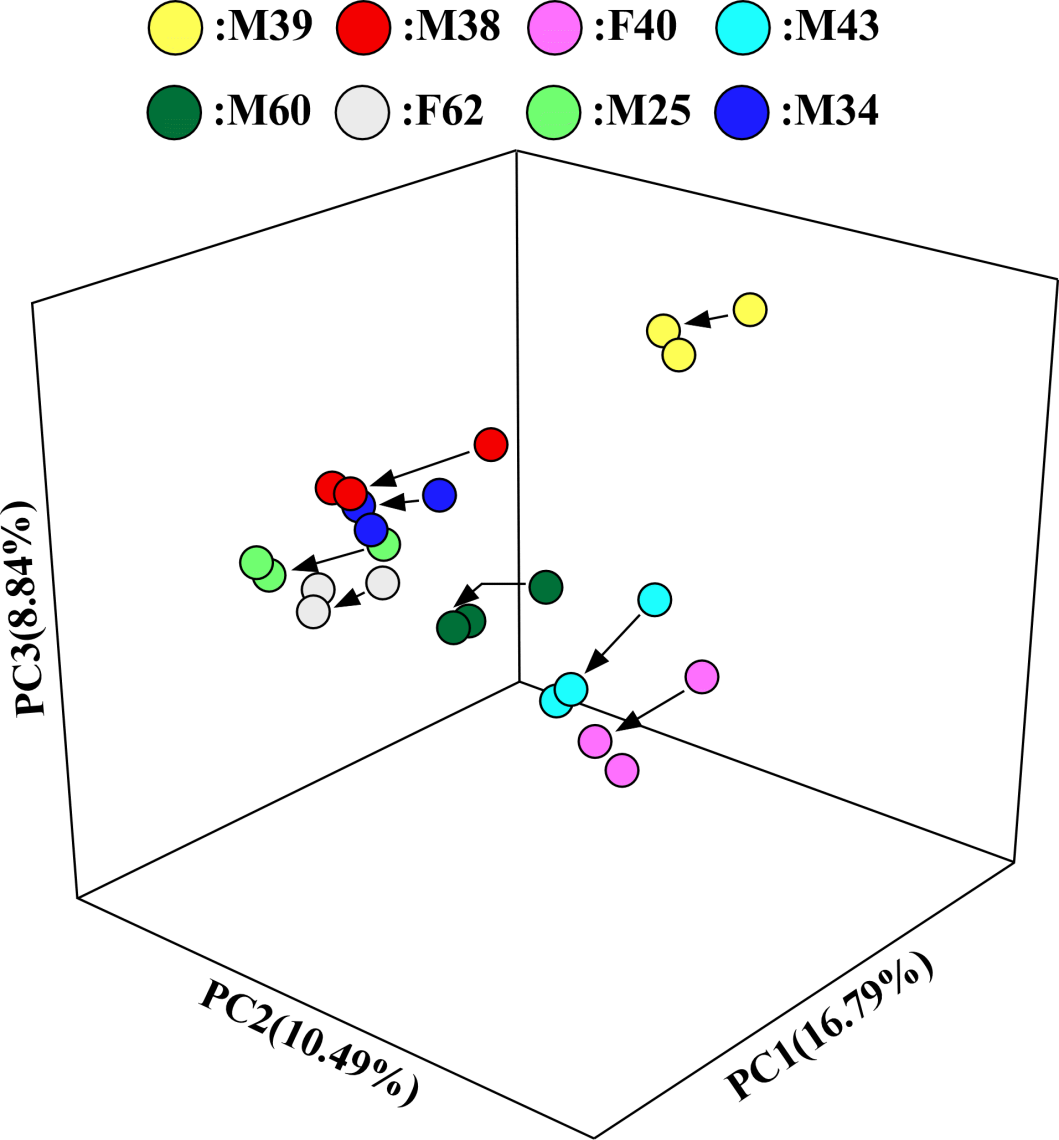
Principal coordinate analysis (PCoA). Samples are color coded as follows: M39, yellow; M38, red; F40, pink; M43, cyan; M60, dark green; F62, gray; M25, green; and M34, blue. Transformations from the original fecal samples to the corresponding KUHIMM culture are shown as arrows. Transformations from the original fecal samples to the corresponding KUHIMM cultures containing taurine are provided but lack an arrow.

At 30 h after the initiation of fermentation, eubacterial copy numbers reached 4.26 × 10^10^ to 9.42 × 10^10^ copies/mL in each vessel of the KUHIMM that had been inoculated using one of the 8 human fecal samples (S1 Table). For comparison, cell densities in feces have been reported to reach as high as 10^11^/wet-g [29]. The microbiota compositions in the original fecal samples and in the respective KUHIMM cultures were analyzed for each of the 8 human subjects by performing bacterial 16S rRNA gene sequencing. Using NGS, an average of 197,509 reads were obtained for each sample. The obtained sequences represented a total of 13 phyla and 164 genera. Bacterial OTU numbers in the original fecal samples ranged from 907–1436 (Fig 2). A total of 688–1360 of these OTUs were not significantly different from the original fecal samples in the respective KUHIMM cultures at 30 h after inoculation (*P* = 0.207), indicating that OTUs in the original fecal samples were maintained during anaerobic cultivation. Similarly, the Shannon-Wiener index values of the original fecal samples (5.022–6.270) were not significantly different from those in the respective cultures after culturing in the KUHIMM (4.397–5.838) (*P* = 0.066). PCoA analysis revealed that the microbiotas in the 8 human subject fecal samples were distinct from one another, but the microbiota of each KUHIMM culture could be assigned to the same cluster as that contained in the respective source (inoculum) fecal samples (Fig 3).

The compositions of microbiota that developed in the KUHIMM cultures supplemented with taurine were examined. OTU numbers and Shannon-Wiener indexes of the KUHIMM cultures containing taurine did not differ significantly from those of the KUHIMM cultures lacking supplemental taurine (*P* = 0.958 and *P* = 0.958, respectively). In addition, PCoA analysis revealed that the microbiota in the KUHIMM cultures containing taurine could be assigned to the same cluster as the respective cultures grown without supplemental taurine (Fig 3).

### Microbiota composition in the fecal samples and single-batch fermentation system with and without taurine

**Fig 4:**
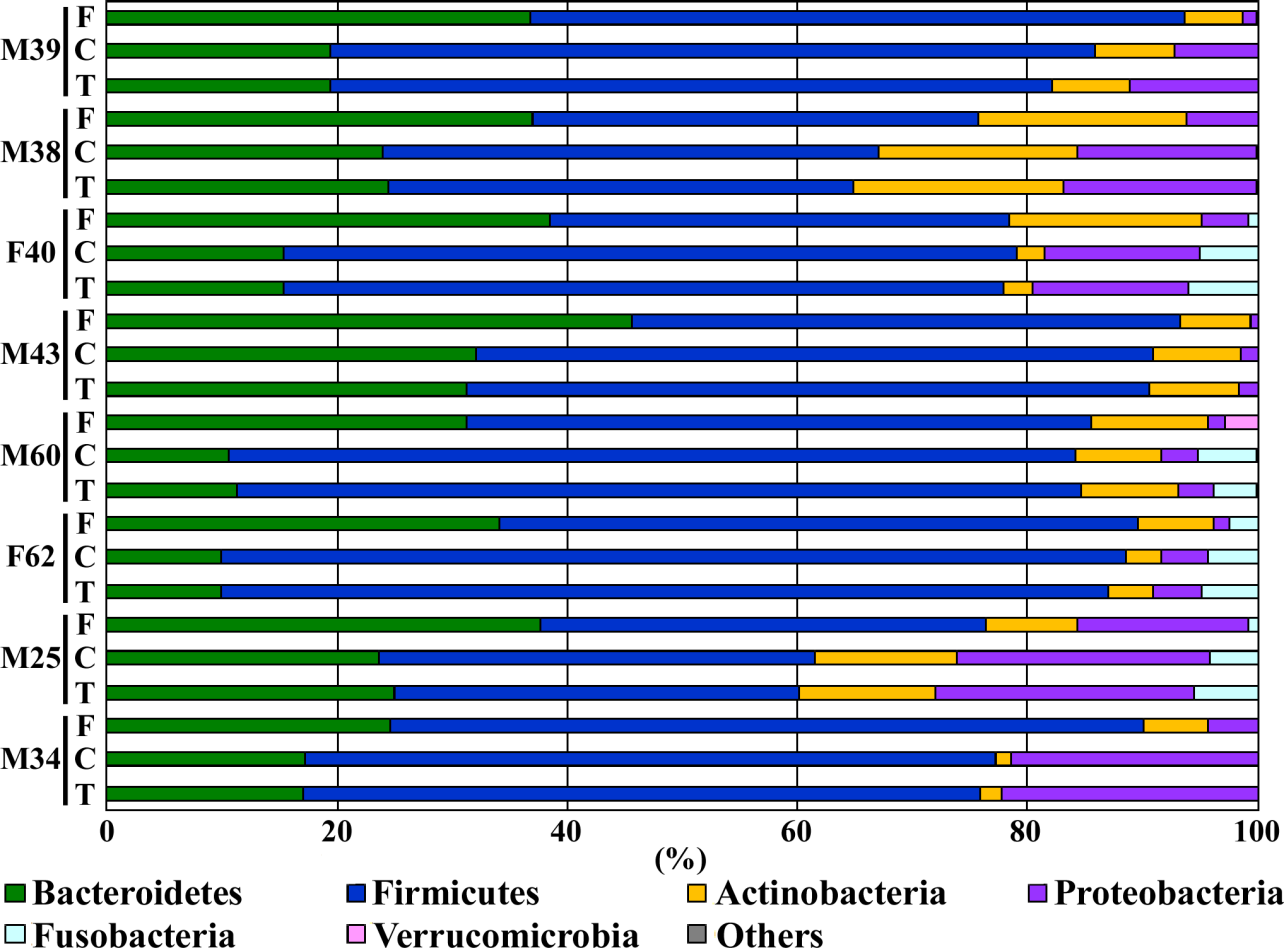
Phylum-level compositional view of bacteria in the eight human fecal samples used as inocula (“F”), in corresponding KUHIMM cultures at 30 h after the initiation of fermentation (“C”), and in corresponding KUHIMM cultures containing taurine at 30 h after the initiation of fermentation (“T”). Individual fecal sample sources are designated as follows: M39 (male, age 39), M38 (male, age 38), F40 (female, age 40), M43 (male, age 43), M60 (male, age 60), F62 (female, age 62), M25 (male, age 25), and M34 (male, age 34). A compositional view of bacterial phyla based on the taxonomic assignment of 16S rRNA genes is shown. Bacterial composition of each sample was estimated based on the results of the RDP classifier.

Approximately 99% of the total bacterial abundance in the samples and cultures could be classified into one of six phyla (Bacteroidetes, Firmicutes, Actinobacteria, Proteobacteria, Fusobacteria, and Verucomicrobia; Fig 4), with the phyla Firmicutes and Bacteroidetes constituting the vast majority of organisms in both the original fecal samples and the respective KUHIMM cultures. However, the phylum Proteobacteria increased its relative abundance in the respective KUHIMM cultures, compared to the original fecal samples. The most abundant genera from the original fecal samples were *Bacteroides* (for M38, F40, F62, and M34), *Prevotella* (for M39, M43, and M60), and *Megamonas* (for M25 with abundance exceeding that of *Bacteroidetes*); the relative abundances of these genera were maintained in the corresponding KUHIMM cultures (*P* = 0.227, 0.372, and 0.958, respectively) (S1 Fig). In addition, the genera *Parabacteroides*, *Blautia*, *Roseburia*, *Faecalibacterium*, *Ruminococcus*, *Megasphaera*, *Phascolarctobacterium*, *Catenibacterium*, *Eubacterium*, *Bifidobacterium*, *Collinsella*, *Sutterella*, and *Fusobacterium* in the original fecal samples were maintained in the corresponding KUHIMM cultures (*P* = 0.637, 0.104, 0.052, 0.083, 0.793, 0.713, 0.227, 0.450, 0.318, 0.128, 0.875, 0.637, and 0.226, respectively). However, an increase in relative microbial abundance was observed for genus *Escherichia* (*P* = 0.041) following culturing. Relative abundances of five microbial phyla (Bacteroidetes, Firmicutes, Actinobacteria, Proteobacteria, and Fusobacteria) (*P*>0.636) and twenty genera (*Bacteroides*, *Parabacteroides*, *Prevotella*, *Streptococcus*, *Blautia*, *Roseburia*, *Faecalibacterium*, *Ruminococcus*, *Acidaminococcus*, *Megamonas*, *Megasphaera*, *Phascolarctobacterium*, *Catenibacterium*, *Eubacterium*, *Bifidobacterium*, *Collinsella*, *Sutterella*, *Escherichia*, *Fusobacterium*, and *Akkermansia*) (*P*>0.563) in the KUHIMM cultures supplemented with taurine were similar to those in cultures not supplemented with taurine (Fig 4 and S1 Fig).

### Effect of taurine on production of SCFA

**Fig 5:**
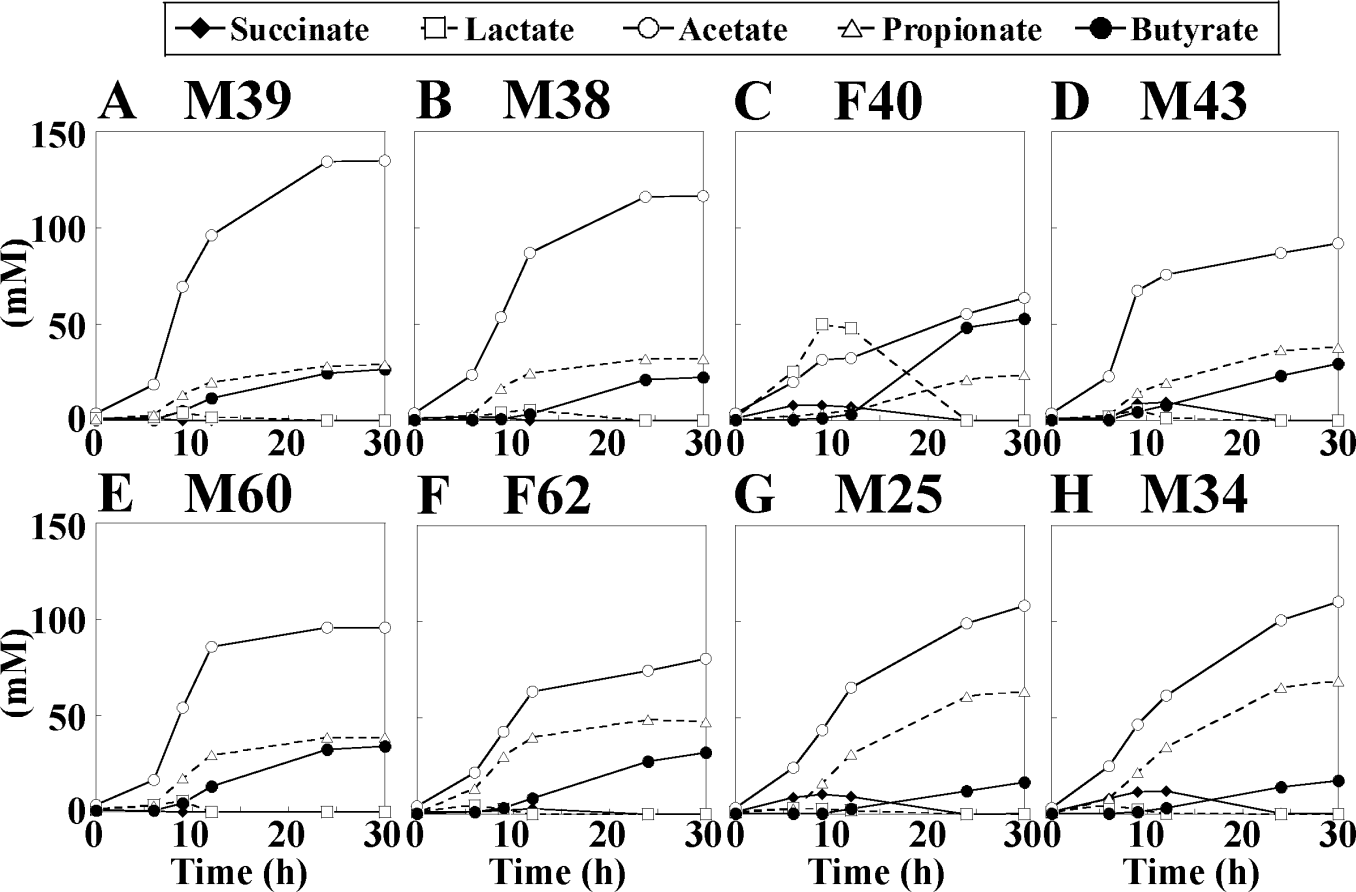
Time-dependent changes of short-chain fatty acid (SCFA) concentrations in the KUHIMM cultures inoculated with one of each of the human fecal samples (A: M39, B: M38, C: F40, D: M43, E: M60, F: F62, G: M25, H: M34).

Gut bacteria in the cecum and large intestine produce SCFAs primarily from nondigestible carbohydrates that pass unaffected through the small intestine [30]. Time-dependent changes of SCFAs were observed in the KUHIMM cultures (Fig 5). For all subjects, levels of acetate, propionate, and butyrate gradually increased to more than 140 mM and were the major SCFA constituents (with acetate the most abundant SCFA) at 30 h after inoculation. Nearly equimolar levels of propionate and butyrate were produced at 30 h after inoculation with M39, M38, M43, or M60. For F40, lactate was the primary SCFA product during the initial phase of fermentation. SCFA profiles were similar in the KUHIMM cultures started from a given inoculum, whether grown in the presence or absence of taurine (S2 Fig).

### Utilization of taurine by microbiota under aerobic conditions

*Escherichia coli* is able to utilize taurine as source of sulfur only under aerobic conditions, with the TauABC and TauD proteins involved in the uptake and metabolism of this compound [26,31,32]. We hypothesized that taurine might be utilized in the KUHIMM cultures grown under aerobic conditions, despite our demonstration (above) that most of the taurine is not utilized by KUHIMM cultures grown under anaerobic conditions. To test this hypothesis, we used 4 of the human fecal samples (M39, M38, F40, and M34) to inoculate KUHIMM cultures grown under aerobic conditions in the presence of 10 mM taurine. About 83% of the added taurine had been consumed at 30 h after inoculation (Fig 1). Thus, taurine was consumed by the same fecal microbiota grown under aerobic conditions.

## Discussion

The NGS analysis revealed that KUHIMM is capable of reproducing the diversity and composition of the microbiota found in human intestinal tracts, where members of the phyla Firmicutes and Bacteroidetes typically dominate [33,34]. In addition, the PCoA indicated that the KUHIMM reproduced the unique individual composition of microbiota from each fecal donor. Furthermore, HPLC analyses confirmed that KUHIMM reproduced the composition of SCFAs in the large intestine, an environment that is characterized by the predominance of acetate, followed by propionate and butyrate [35]. Our previous study successfully demonstrated that the bifidogenic properties of the oligosaccharides were reproduced in the KUHIMM, an observation consistent with reports from other studies [36,37]. This evidence indicates the reliable capacity of the KUHIMM for simulating individual human colonic microbiota, thereby allowing us to evaluate the fate of dietary taurine and speculate on its impact on human health as follows.

Despite its broad distribution in the human body, taurine is not regarded as an essential amino acid because the compound is not required for protein synthesis. Nevertheless, taurine has been long known to play a range of significant roles in the host’s physiology and biochemistry, thereby contributing to human health [1]. Taurine thus has been increasingly used as one of the most promising dietary supplements, capable of providing numerous health benefits [38–40]. Additionally, several tissue culture-based studies and animal experiments have suggested that taurine has an ameliorating effect against inflammatory bowel diseases (IBDs) involving the human large intestine, including ulcerative colitis and Crohn's disease [9,41,42]. However, most, if not all, dietary taurine is normally absorbed in the small intestine, which is equipped with a taurine-specific uptake system [43]. As a result, it is difficult for taurine to reach the large intestine to exert anti-inflammatory effects. Moreover, we initially assumed that even if some quantity of taurine reached the large intestine, the compound would be readily metabolized by members of the colonic microbiota. For example, Backus et al. [44] showed that taurine was degraded in anaerobic cultures initiated using cat feces as the inoculum. However, as we demonstrated in the present study, the majority of the taurine added to anaerobic KUHIMM cultures initiated from each of 8 human subjects remained intact even after a 30-h-long incubation. Furthermore, we found that taurine had no effect on the microbiota composition at the phylum and genus levels. This result contrasts to that reported in mice, where supplementation with taurine yielded feces with a reduced proportion of the phylum Proteobacteria [11]. This discrepancy may be ascribed to the difference in microbiota between human and other animals, as demonstrated previously [13,14]. Alternatively, the discrepancy may be simply due to the markedly higher dose of dietary taurine (165 mg/kg) in the mouse study [11] compared to the recommended dose for humans (ca. 40 mg/kg [3 g for 70 kg adult]) [20] that was used in the present study. Recently, Rosa et al. [45] demonstrated that taurine supplementation (3 g per day) significantly reduced several markers of inflammation in human subjects while Ward at al. [46] reported that a concentration of taurine as low as 10 nM had anti-inflammatory effects *in vitro*. These findings, and the results of the present study, suggest that a trace amount of dietary taurine might escape absorption in the small intestine (in some instances, via a drug delivery system designed to carry taurine to the large intestine without being absorbed in the small intestine), reach the large intestine without being degraded by the human microbiota (in contrast to the feline microbiota [44]), and exert anti-inflammatory effects. Interestingly, we found that most of the supplemental taurine was degraded when cultured aerobically with the human fecal samples. Eichhorn and colleagues [26] demonstrated that *E*. *coli*, one of the common bacteria in the large intestinal microbiota, imports and utilizes taurine when cultured under aerobic conditions, implying that microbial degradation of taurine may require aerobic conditions. In the present study, we utilized culture vessels and did not consider the effect of taurine on the host immune system. Taurine reportedly activates the intestinal inflammasome to induce downstream generation of anti-microbial peptides, contributing to the construction of a symbiotic intestinal microenvironment [47]. Thus, in the actual humane colon, taurine would indirectly affect microbiota composition.

## Supporting information

S1 Fig Genus-level compositional view of bacteria in the 8 human fecal samples used as inocula (“F”), in corresponding KUHIMM cultures at 30 h after the initiation of fermentation (“C”), and in corresponding KUHIMM cultures containing taurine at 30 h after the initiation of fermentation (“T”). Individual fecal sample sources are designated as follows: M39 (male, age 39), M38 (male, age 38), F40 (female, age 40), M43 (male, age 43), M60 (male, age 60), F62 (female, age 62), M25 (male, age 25), and M34 (male, age 34). ‘Others’ includes unclassified genera and those that represented less than 3% of the total numbers.
(TIF)

S2 Fig Time-dependent changes of short-chain fatty acid (SCFA) concentrations in the KUHIMM cultures supplemented with taurine and inoculated with one of each of the human fecal samples (A: M39, B: M38, C: F40, D: M43, E: M60, F: F62, G: M25, H: M34).
(TIF)

S1 Table 16S rRNA gene copy numbers of eubacteria. The KUHIMM culture was sampled at 30 h after the initiation of fermentation.
(DOCX)
