# Supplementary material for: Taurine does not affect the composition, diversity, or metabolism of human colonic microbiota simulated in a single-batch fermentation system
Source: PLoS One. 2017 Jul 10;12(7):e0180991. doi: 10.1371/journal.pone.0180991 (PMC5507302; doi:10.1371/journal.pone.0180991)
Supplement: S1 Table — The KUHIMM culture was sampled at 30 h after the initiation of fermentation. (DOCX) [file pone.0180991.s003.docx]

**S1 Table. 16S rRNA gene copy numbers of eubacteria.** The KUHIMM culture was sampled at 30 h after the initiation of fermentation.

| Human subject | Eubacteria |
| --- | --- |
|  | ×10^10^ (copies/mL) |
| M39 | 6.49 |
| M38 | 4.26 |
| F40 | 8.24 |
| M43 | 5.89 |
| M60 | 6.44 |
| F62 | 7.34 |
| M25 | 9.42 |
| M34 | 8.62 |
